# Paclitaxel Inhibits KCNQ Channels in Primary Sensory Neurons to Initiate the Development of Painful Peripheral Neuropathy

**DOI:** 10.3390/cells11244067

**Published:** 2022-12-15

**Authors:** Zizhen Wu, Gabor Toro, Guoying Xu, Danny Dang, Charmaine Prater, Qing Yang

**Affiliations:** Department of Neurobiology, University of Texas Medical Branch, Galveston, TX 77555, USA

**Keywords:** KCNQ channels, DRG, paclitaxel, pain, neuropathy

## Abstract

Cancer patients undergoing paclitaxel infusion usually experience peripheral nerve degeneration and serious neuropathic pain termed paclitaxel-induced peripheral neuropathy (PIPN). However, alterations in the dose or treatment schedule for paclitaxel do not eliminate PIPN, and no therapies are available for PIPN, despite numerous studies to uncover the mechanisms underlying the development/maintenance of this condition. Therefore, we aimed to uncover a novel mechanism underlying the pathogenesis of PIPN. Clinical studies suggest that acute over excitation of primary sensory neurons is linked to the pathogenesis of PIPN. We found that paclitaxel-induced acute hyperexcitability of primary sensory neurons results from the paclitaxel-induced inhibition of KCNQ potassium channels (mainly KCNQ2), found abundantly in sensory neurons and axons. We found that repeated application of XE-991, a specific KCNQ channel blocker, induced PIPN-like alterations in rats, including mechanical hypersensitivity and degeneration of peripheral nerves, as detected by both morphological and behavioral assays. In contrast, genetic deletion of KCNQ2 from peripheral sensory neurons in mice significantly attenuated the development of paclitaxel-induced peripheral sensory fiber degeneration and chronic pain. These findings may lead to a better understanding of the causes of PIPN and provide an impetus for developing new classes of KCNQ activators for its therapeutic treatment.

## 1. Introduction

Most patients with breast, ovarian, or lung cancer receive paclitaxel (Taxol^®^)-based chemotherapy [1]. Paclitaxel is so effective in cancer treatment that it is now included in the “WHO list of Essential Medicines” (WHO, Geneva, Switzerland, 2016). However, the sensory peripheral neuropathy that is often associated with neuropathic pain after paclitaxel perfusion, termed paclitaxel-induced peripheral neuropathy (PIPN), is common and severe. The incidence and severity of PIPN is duration and dose-related such that nearly 88% of patients receiving paclitaxel manifest symptoms [2], resulting in reduced overall quality of life; in some instances, this leads to treatment suspension, which negatively impacts survival [2].

Despite its public health importance, the pathogenesis of PIPN is not well understood. Paclitaxel is known to stabilize microtubules, blocking mitosis and promoting cell death [1]; moreover, it binds to the β-tubulin of axonal microtubules, interfering with axonal transport [3,4,5,6]. Recent studies have demonstrated that paclitaxel disrupts the axonal transport of the mRNA encoding components of the mitochondrial fusion/fission machinery that controls mitochondrial dynamics [7,8], resulting in altered mitochondrial morphology and impaired mitochondrial function [7,9,10,11]. The interplay between mitochondria and oxidative stress can further compromise the survival and function of sensory neurons [12,13,14,15]. However, impairing microtubule assembly in vivo with TZT-1027, a derivative of dolastatin [16], failed to induce neuropathological alterations and chronic pain [17]. Additionally, both acetyl-L-carnitine (a mitoprotective drug) and glutathione (an antioxidant) failed to prevent PIPN in large clinical trials [18,19]. Thus, there must be an unexplored factor that works with the currently proposed mechanisms to induce PIPN.

Several clinical trials have revealed that cancer survivors usually have a high risk of developing peripheral neuropathy and chronic neuropathic pain if they experience acute pain during paclitaxel infusion [20,21], suggesting a link between the acute hyperexcitation of primary sensory neurons and the development of PIPN. Paclitaxel induces the immediate excitation of dorsal root ganglion (DRG) neurons isolated from human tissue samples [22], as well as acute peripheral and visceral nociception in vertebrate animals [23]. However, the mechanism underlying such paclitaxel-induced acute effects is unknown. Our previous study indicates that decreasing neuronal excitability by systematic application of a KCNQ channel opener prevents the development of PIPN [24]. However, this study gives no clue as to whether paclitaxel-induced acute neuronal excitation results from alterations in the activity of KCNQ channels or other ion channels, and whether the protective effect results from decreased excitability of the peripheral or central nervous system.

In this study, we aim to investigate the molecular mechanisms underlying paclitaxel-induced acute excitation of DRG neurons and its contribution to the development of PIPN. By combining a transgenic approach and electrophysiological recordings, we provide direct evidence that acute increases in the excitability of peripheral sensory neurons resulting from paclitaxel-induced inhibition of KCNQ channels initiates PIPN chronic alterations. This study thus leads to a better understanding of the mechanism underlying PIPN as well as delineating novel targets related to the neurotoxicity of paclitaxel.

## 2. Materials and Methods

Male, adult, Sprague-Dawley rats (200–300 g) were purchased from Charles River Laboratories (Wilmington, MA, USA). Rats were housed 2 per cage in a controlled environment (12 h reversed light/dark cycle, 21 ± 1 °C) with standard food and water. The rats were allowed to adjust to their environment for a week before the experiment.

### 2.1. Mouse Strains and Genotyping

To generate KCNQ2 flox mice, we purchased Kcnq2^tm1a(EUCOMM)Wtsi^ embryonic stem (ES) cells from the International Mouse Phenotyping Consortium. The ES cells were used to generate germline-transmitting chimeras, who were crossed with FLPeR mice (from the Jackson Laboratory, Bar Harbor, ME, USA) to remove the frt-flanked Laz-neomycin cassette. The following primers were used to genotype the KCNQ2flox allele: 5′-GGGAGAGGGAAACAGAGAGG-3′ and 5′-AAAGAACCAACTCCACGCTG-3′. Mice expressing two copies of LoxP-flanked alleles are here referred to as KCNQ2^flx/flx^ mice. Advillin-Cre mice were kindly provided by Dr. Fan Wang [25]. The KCNQ2^flx/flx^ mice were crossed with the Advillin-Cre mice to generate conditional knockout animals, which are referred to as KCNQ2^flx/flx^//Advillin-Cre or KCNQ2*cko* mice in this manuscript. Both male and female KCNQ2*cko* mice were used, and no obvious behavioral and morphological differences between the sexes were noted. The data from both sexes were thus analyzed together. In all cases, a Cre+ male was crossed with a Cre- female to ensure Cre transmission only from the male progenitor [25].

### 2.2. Administration of Paclitaxel

Paclitaxel (TEVA Pharmaceutical Inc., North Wales, PA, USA) was delivered intraperitoneally (i.p., 2 mg/kg in 0.9% saline, once daily) on days 1, 3, 5, and 7 (8:00 to 10:00 a.m.) [26]. Sham animals received an equal gradient of cremophor EL (Sigma, St. Louis, MO, USA) and ethanol of the same volume as that used in the paclitaxel group, which were administered at the same time that the experimental mice received paclitaxel (days 1, 3, 5, and 7, 8:00 to 10:00 a.m.).

### 2.3. Behavioral Tests

Animals were tested in the light phase. Animals underwent testing before and after paclitaxel administration on the indicated day/time. The staff who performed the behavioral tests were blinded to the genotypes of the mice and the drug treatments. After habituation of each animal in the testing chamber for either 20 min (for SD rats) or 60 min (for mice), a series of calibrated *von Frey* filaments (Stoelting, Wood Dale, IL, USA) were pressed against the glabrous surface of the hind paws, and the thresholds of mechanical sensitivity were analyzed with the “up-down” method [27,28]. A conditioned place preference (CPP) device (Med Associates Inc., Fairfax, VT, USA) was used to test spontaneous pain as previously described [29,30]. The CPP device contained two chambers, one white, the other black. On the first day, each animal was placed in the CPP chambers for 30 min of habituation. On the following 3 days, each animal was conditioned with a twice daily conditioning injection of analgesic and vehicle, each of which became associated with a certain chamber. During the anesthetic conditioning session, the animal received retigabine (10 mg/kg, i.p.). Five minutes after injection, the subject was kept in the white chamber for 1 h. In another conditioning session, the same animal was restricted to the black chamber for 1 h following a saline injection (i.p.). On the fifth day, the testing animal (without injection) was placed in the black chamber and allowed to freely access both chambers. The system recorded the time the animal stayed in each of the 2 chambers over a 30 min period.

### 2.4. DRG Neuron Dissociation and Culturing

After an i.p. injection of beuthanasia (75 mg/kg, Merck Animal Health, Kenilworth, NJ, USA), the animals were intracardially perfused with cold phosphate-buffered saline (PBS), and the lumbar column was opened to harvest DRGs from lumbar vertebrae 4 and 5. The ganglia were then minced and incubated for 40 min at 34 °C in Dulbecco’s Modified Eagle Medium (DMEM; Invitrogen, Waltham, MA, USA) containing 0.4 mg/mL trypsin (Worthington, Lakewood, NJ, USA) and 0.6 mg/mL collagenase (Roche, Mannheim, Germany). The cell-containing solution was then centrifuged (300 rpm for 5 min). The isolated cells were plated onto 8 mm cover glasses (Warner Instruments, Hamden, CT, USA) precoated with 50 mg/mL poly-L-lysine; they were then incubated (37 °C, 5% CO_2_) in DMEM without serum for 18–24 h prior to the electrophysiological recordings.

### 2.5. Chinese Hamster Ovary (CHO) Cell Transfection

Human Kcnq2 and Kcnq3 alone or in combination (1:1 ratio) were transiently expressed in CHO cells by transfection using Lipofectamine 2000 (Invitrogen). The cells were dissociated 12 h after transfection, plated onto poly-L-lysine-precoated coverslips and incubated (5% CO_2_, 37 °C) for another 12 h prior to the electrophysiological recordings.

### 2.6. Electrophysiological Recordings of DRG Neurons and CHO Cells

A P-1000 micropipette puller (Sutter Instrument Co., Novato, CA, USA) was used to pull electrodes from borosilicate glass capillaries (BF150-110-10, Sutter Instrument Co., Novato, CA, USA), and electrodes with a resistance of ~2 MΩ were retained for use in the following experiments. Neurons were viewed using an Eclipse Ti inverted microscope (Nikon, Tokyo, Japan). Both whole-cell current clamp and voltage clamp were performed with a MultiClamp 700B amplifier with an Axon Digidata 1550B data acquisition system (Molecular Devices, San Jose, CA, USA). The resting membrane potential (RMP) and any spontaneous firing of DRG neurons were recorded by patching the cells at 0 pA. Both KCNQ-like currents and capsaicin-sensitive currents were recorded under a voltage-clamp model by patching cells at −20 or −60 mV, respectively. KCNQ-like currents in both DRG neurons and CHO cells were recorded by hyperpolarizing the cell from −20 to −60 mV. Signals were sampled at ≥ 10 kHz. The normal pipette solution contained (in mM) 134 KCl, 13.2 NaCl, 1.8 CaCl_2_, 1.6 MgCl_2_, 3 EGTA, 9 HEPES, 0.3 Na-GTP, and 1 Mg-ATP (pH 7.2, 300 mOsM). The BAPTA-containing pipette solution included (in mM) 94 KCl, 13.2 NaCl, 1.6 MgCl_2_, 10 tetrapotassium BAPTA, 9 HEPES, 1 Mg-ATP, and 0.3 Na-GTP (pH 7.2, 300 mOsM). The bath solution included (in mM) 140 NaCl, 3 KCl, 2 MgCl_2_, 1.88 CaCl_2_, 10 HEPES, and 10 glucose (pH 7.4, 320 mOsM). Paclitaxel, XE-991 (Tocris, Bristol, UK), α-dendrotoxin (Alomone labs, Jerusalem, Israel), phrixotoxin-2 (Alomone labs, Jerusalem, Israel), BDS-II (Alomone labs, Jerusalem, Israel), KT10 (Abcam, Waltham, MA, USA), wortmannin (Tocris, Bristol, UK), BAPTA (Life Tech, Carlsbad, CA, USA), U73122 (Tocris, Bristol, UK), and st-Ht31 (Tocris, Bristol, UK) were added to either bath solution (Paclitaxel, XE-991, α-dendrotoxin, phrixotoxin-2, BDS-II) or pipette solution (KT10, wortmannin, BAPTA, U73122, st-Ht31).

### 2.7. Intraepidermal Nerve Fiber Staining and Quantification

Under deep anesthesia (75 mg/kg, euthanasia i.p., Merck Animal Health, Kenilworth, NJ, USA), a 3-mm (for rats) or 2-mm (for mice) glabrous skin biopsy punch was harvested from the hind paws. Tissues were placed in Zamboni’s fixative solution overnight (4 °C) and 20% sucrose for at least another 24 h before embedding in a frozen compound prepared at an optimal cutting temperature for cryostat (25 μm). Transverse sections were permeabilized and blocked with 0.5% Triton X-100 and 5% serum for 1 h. Free-floating samples were then incubated with a combination of anti-collagen IV (Southern Biotech, AL, USA; Cat# 1340) and anti-protein gene product 9.5 (PGP9.5) (Proteintech Group Inc., IL; Cat# 17430) antibodies overnight at 4 °C; the primary antibodies were omitted in negative controls. After 3 rinses in PBS, the samples were incubated with secondary antibodies for 2 h at room temperature; labeled samples were cover-slipped with Vectashield (Vector Labs) following washing in PBS. We used a Nikon confocal microscope (Nikon, Tokyo, Japan) to image the sections.

ImageJ software (NIH) was used to quantify the IENFs within a field of view as previously described [24]. Four to five slices from each subject were randomly selected. For each imaged slice, peripheral nerve fibers (stained with PGP9.5) that crossed the collagen IV-stained dermal/epidermal junction and the length of the epidermis within three fields of view were analyzed. IENF density was calculated as the total number of fibers per unit length of epidermis (IENF/mm). The staff member who performed IENF staining and counting was blinded to the genotypes of the mice and the treatment conditions.

### 2.8. Statistics

Transgenic mice were genotyped and assigned to experimental or control groups by a separate individual who was not involved in conduct of the experiments. Reagents for the experiments were prepared and numbered by an individual not involved in the direct conduct of the experiment. Animals were randomly assigned to different groups. All behavioral testing and histological experiments were blindly performed. Un-blinding occurred only after all data collection, data-entry, and database formatting were finished. Both Sigmaplot (version 14, Systat software, San Jose, CA, USA) and Prism (version 9.0, GraphPad, La Jolla, CA, USA) were used for data analysis. All data are presented as the mean ± S.E.M. or percentage. The α level for all statistical tests was 0.05. One-way or two-way ANOVA followed by Tukey’s multiple comparison tests were used to compare more than two groups. The correlation of DRG neuron responses for different blockers was tested using Spearman’s test. Two-tailed unpaired or paired *t* tests were used to compare results obtained before and after treatment in the same cohort. Results with *p* values of <0.05 were considered statistically significant (*, *p* < 0.05; **, *p* < 0.01; ***, *p* < 0.001). Detailed statistical information for all experiments is presented in the figure legends.

## 3. Results

### 3.1. KCNQ Channels Are Involved in the Paclitaxel-Induced Excitation/Depolarization of DRG Neurons

**Figure 1:**
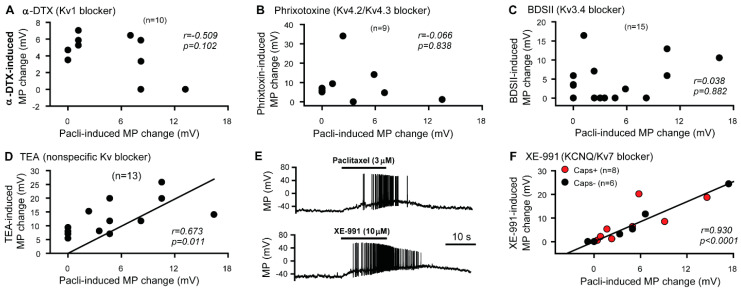
Relationship between the paclitaxel- and Kv channel blocker-induced membrane depolarization of dorsal root ganglion (DRG) neurons. (**A**) Membrane potential changes induced by 100 nM α-dendrotoxin and 3 μM paclitaxel. *r* = −0.509, *p* = 0.102, Spearman rank order test. (**B**) Membrane potential changes induced by 1 μM phrixotoxin-2 and 3 μM paclitaxel. *r* = 0.066, *p* = 0.838, Spearman rank order test. (**C**) Membrane potential changes induced by 1 μM BDS-I (blood-depressing substance-I) and 3 μM paclitaxel. *r* = 0.038, *p* = 0.882, Spearman rank order test. (**D**) Membrane potential changes induced by 25 mM TEA and 3 μM paclitaxel. *r* = 0.673, *p* = 0.011, Spearman rank order test. (**E**) Representative traces showing XE-991- and paclitaxel-induced membrane depolarization and cell firing in the same cell. (**F**) Membrane potential changes induced by 10 μM XE-991 and 3 μM paclitaxel. *r* = 0.930, *p* < 0.0001, Spearman rank order test. Capsaicin-sensitive cells are indicated as red dots. Each filled dot in the plots represents one neuron. N in each panel indicates the number of DRG neurons patched. MP, membrane potential; Pacli, paclitaxel; Caps, capsaicin.

Because the activity of voltage-gated potassium channels (Kv) plays a pivotal role in sensory neuron excitation, we investigated whether any of these channels are involved in the paclitaxel-induced depolarization/hyperexcitability of DRG neurons. First, we investigated the relationship between the depolarization induced by various K^+^ channel blockers and that induced by paclitaxel using whole-cell current-clamp (0 pA) in DRG neurons dissociated from naive rats. Our data indicate that the depolarization induced by 100 nM α-dendrotoxin (a specific Kv1 blocker) [31], 1 μM phrixotoxin-2 (a specific Kv4.2 and Kv4.3 blocker) [32], or 1 μM BDS-II (a specific Kv3.4 blocker) [33] was not correlated with the depolarization induced by 3 μM paclitaxel in the same DRG cells following washout of the previous channel blocker with locally delivered bath solution (Figure 1A–C). However, tetraethylammonium (TEA), a nonspecific K^+^ channel blocker that blocks KCNQ channels [34,35,36,37], and XE-991, a specific KCNQ (Kv7 family) channel blocker [35,38], depolarized primary sensory neurons, both capsaicin-sensitive and capsaicin-insensitive, in a manner that was significantly correlated with the response induced by 3 μM paclitaxel in vitro (Figure 1D–F). These data strongly suggest that paclitaxel inhibits KCNQ channels in DRG neurons.

**Figure 2:**
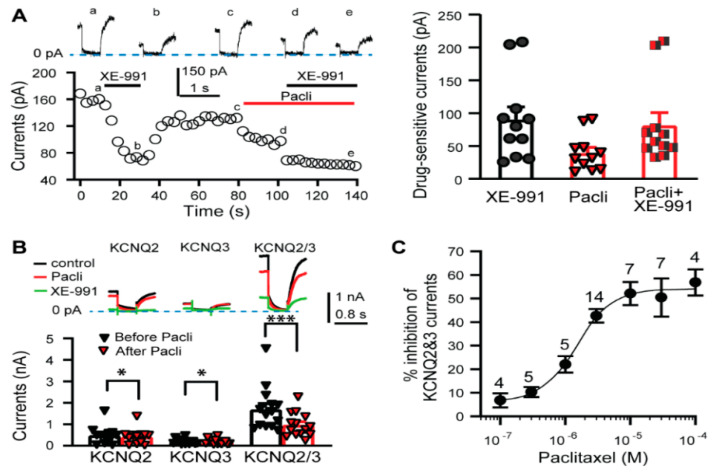
Paclitaxel inhibits KCNQ currents in isolated DRG neurons and Chinese hamster ovary (CHO) cells. (**A**) Representative traces and time course (left panel), as well as summary data (right panel), showing the effect of 10 μM XE-991, 3 μM paclitaxel, and their combination on the currents induced by hyperpolarization from −20 to −60 mV in isolated DRG neurons. Filled symbols in the bar graph represent individual DRG neurons. XE-991-sensitive currents = a − b, Pacli-sensitive currents = c − d, Pacli + XE-991-sensitive currents = c − e. *p* = 0.3405, F_(2,30)_ = 1.117. One-way ANOVA used to compare the data in the bar graphs. (**B**) Current traces (upper panel) and bar graph (bottom panel) showing 3 μM paclitaxel-induced inhibition of XE-991 (10 μM)-sensitive currents in CHO cells overexpressing KCNQ2, KCNQ3, or KCNQ2/3 (1:1). Filled symbols in the bar graph represent individual CHO cells. *p* < 0.0001, t(11) = 3.025 for KCNQ2; *p* = 0.0387, t(13) = 2.3 for KCNQ3; *p* = 0.0002, t(13) = 5.266 for KCNQ2/3. Paired *t* test (two-tailed) used to compare the data in the bar graphs. (**C**) Scatter plot showing the effects of different concentrations of paclitaxel on the XE-991-sensitive current of CHO cells overexpressing KCNQ2/3 (1:1). The number above each point in the graph is the number of patched cells. Data are represented as mean ± SEM. *, *p* < 0.05; ***, *p* < 0.001. Pacli, paclitaxel.

To directly investigate whether paclitaxel inhibits KCNQ channels, we then performed whole-cell voltage-clamping with DRG neurons isolated from naïve rats. While both XE-991 and paclitaxel inhibited currents induced by a standard protocol (hyperpolarization from −20 to −60 mV) in isolated DRG neurons, the inhibition induced by 10 μM XE-991 (90.45 ± 19.1 pA) was comparable to that induced by the combination of 3 μM paclitaxel and 10 μM XE-991 (81.7 ± 19.1 pA) (Figure 2A). These findings indicate that paclitaxel-inhibited currents in isolated DRG neurons are XE-991-sensitive, suggesting these currents are mediated by KCNQ channels.

KCNQ channels assemble as tetramers of five Kv7 members (KCNQ1-KCNQ5, also known as Kv7.1-Kv7.5) [39]; several of these members, including KCNQ2, KCNQ3 and KCNQ5, are expressed in rat DRG neurons, including capsaicin-sensitive nociceptors [40,41]. Notably, the KCNQ currents mediated by KCNQ2 homomultimers or heteromultimers (KCNQ2/3) [34,35,36,37], but not those mediated by KCNQ5 homomultimers [42], are highly sensitive to TEA and XE-991. Thus, to detect whether paclitaxel inhibits KCNQ2 or KCNQ3 channels, we overexpressed KCNQ2, KCNQ2/3, or KCNQ3 in Chinese hamster ovary (CHO) cells, in which endogenous KCNQ channels are undetectable [43]. Our electrophysiological recordings showed that the currents induced by a hyperpolarization protocol (from −20 to −60 mV) in these cells were XE-991 sensitive, since 10 μM XE-991 almost eliminated the currents (Figure 2B, upper panel). The inhibition induced by 3 μM paclitaxel was much stronger in KCNQ2/3-overexpressing cells (42.68 ± 2.87%) than in KCNQ2-overexpressing cells (16.02 ± 3.90%) or KCNQ3-overexpressing cells (3.82 ± 1.91%) (Figure 2B, bottom panel). Furthermore, the inhibitory effect of paclitaxel on CHO cells overexpressing KCNQ2/3 was dose dependent; at a threshold concentration of 0.1 µM, paclitaxel began inhibiting the XE-991-sensitive current, and maximal inhibition occurred at approximately 10 μM (Figure 2C). This experiment further confirmed that paclitaxel inhibits KCNQ channels.

### 3.2. Paclitaxel Fails to Induce Acute Neuronal Excitation in KCNQ2 Conditional Knockout Mice

Although KCNQ3 can form homomeric channels in Kcnq2 mutant mice [44], paclitaxel had a very weak effect on KCNQ3 channels (Figure 2). Thus, we generated KCNQ2^flx/flx^//Advillin-Cre+ mice, also called KCNQ2 conditional knockout mice (KCNQ2*cko*), in which Kcnq2 is specifically deleted from peripheral sensory neurons, as Advillin is exclusively expressed in sensory neurons [25].

**Figure 3:**
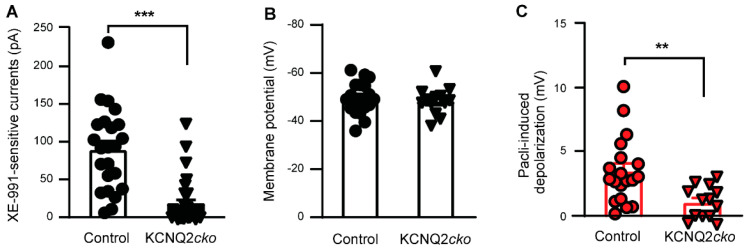
Effect of KCNQ2 deletion from primary sensory neurons on the paclitaxel- and XE-991-induced response. (**A**) XE-991 (10 μM)-sensitive currents in DRG neurons from control and KCNQ2*cko* mice. *p* < 0.001, t(52) = 6.181, unpaired *t* test (two-tailed). Black symbols in columns represent one DRG neuron each. (**B**) Resting membrane potential of DRG neurons from control and KCNQ2cko mice. *p* = 0.593, t(30) = 0.541, unpaired *t* test (two-tailed). Black symbols in columns represent one DRG neuron each. (**C**) Paclitaxel (3 μM)-induced membrane depolarization of DRG neurons from control and KCNQ2*cko* mice. *p* = 0.0038, t(30) = 3.137, unpaired *t* test (two-tailed). Filled symbols in columns represent one DRG neuron each. **, *p* < 0.01; ***, *p* < 0.001.

We first verified the deletion of KCNQ2 from DRG neurons by electrophysiological recordings. Robust XE-991-sensitive currents were recorded in DRG neurons from control mice following hyperpolarization from −20 to −60 mV [45]; however, no such currents were detected in the majority of patched DRG neurons dissociated from KCNQ2*cko* mice (Figure 3A). We also investigated the effect of paclitaxel on the membrane potential of DRG neurons dissociated from KCNQ2*cko* mice, with age-matched Kcnq2^flx/flx^ mice serving as controls. Similar to the results of a previous report [44], the baseline membrane potential of DRG neurons in the KCNQ2*cko* mice was not significantly different from that of the control mice (48.34 ± 1.58 vs. 49.57 ± 1.52 mV) (Figure 3B); however, the paclitaxel-induced depolarization was significantly lower in magnitude in the DRG neurons of KCNQ2*cko* mice than in those of the control group (Figure 3C). These data confirmed that KCNQ2 within sensory neurons mediates paclitaxel-induced acute excitation.

### 3.3. PIP2 Is Involved in the Paclitaxel-Induced Inhibition of KCNQ Channels

**Figure 4:**
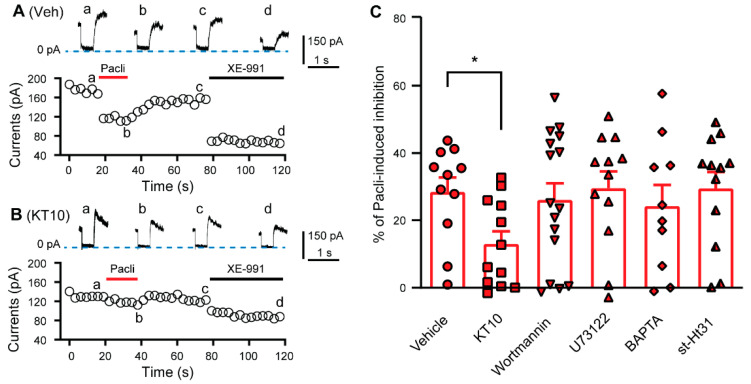
Effect of PIP2 and associated signaling pathways on paclitaxel-induced inhibition of XE-991-sensitive currents. (**A**) Current traces and time course showing the effect of paclitaxel (3 μM) and XE-991 (10 μM) on the elicited currents of DRG neurons with normal intracellular solution in the recording pipette. The neuron was hyperpolarized from −20 to −60 mV for 500 ms. Paclitaxel-sensitive currents = a − b. (**B**) Current traces and time course showing the effects of paclitaxel (3 μM) and XE-991 (10 μM) on elicited currents of DRG neurons when PIP2 antibody (KT10, 20 mg/mL) was included in the recording pipette. The neuron was hyperpolarized from −20 to −60 mV for 500 ms. Paclitaxel-sensitive currents = a − b. (**C**) Summary data showing the effect of KT10 (20 mg/mL), U73122 (1 μM), wortmannin (20 nM), BAPTA (10 mM), and st-Ht31 (50 μM) on paclitaxel-induced inhibition of hyperpolarization-induced currents of DRG neurons ((a-b)/a). Filled symbols in the bar graph represent individual DRG neurons. Control vs. KT10, *p* = 0.0121, t(21) = 2.745; Control vs. U73122, *p* = 0.8633, t(21) = 0.1743; Control vs. wortmannin, *p* = 0.7366, t(25) = 0.3401; Control vs. BAPTA, *p* = 0.5771, t(19) = 0.5674; Control vs. st-Ht31, *p* = 0.8741, t(21) = 0.1604. Unpaired *t* test, two tailed. *, *p* < 0.05. Pacli, paclitaxel.

KCNQ channels are gated by phosphatidylinositol 4,5-bisphosphate (PIP2) [45]. Thus, we tested whether PIP2 is necessary in the paclitaxel-induced inhibitory effect. As PIP2 antibody (KT10) reduces the level of PIP2 in neuronal membranes [46], we compared the effect of KT10 (20 μg/mL) in the recording pipette solution on paclitaxel-induced inhibition on XE-991-sensitive currents with that of a normal pipette solution. Currents were produced in dissociated DRG neurons isolated from naive rats by hyperpolarizing the cells from −20 to −60 mV. The cells were exposed to 3 μM paclitaxel for 20 s followed by washout and another exposure to XE-991 (10 μM). Recordings obtained using the KT10-containing pipette solution showed baseline currents that were lower than those obtained using a normal pipette solution (from 147.65 ± 15.18 to 83.5 ± 14.11 pA), and the percentage of paclitaxel-sensitive currents was also significantly attenuated (Figure 4). These data suggest that PIP2 is necessary for the paclitaxel-induced inhibition of KCNQ channels in DRG neurons.

Notably, phosphatidylinositol-3-kinase (PI3K) activation decreases local PIP2 levels [45], and paclitaxel activates the PI3K signaling pathway [47], and serves to bind a Ca^2+^-binding protein involving PIP2 trafficking, neuronal Ca^2+^ sensor-1 (NCS-1) [48,49,50]. Thus, to investigate whether PI3K and NCS-1 are involved in the paclitaxel-induced inhibition of KCNQ channels in DRG neurons, we included wortmannin (20 nM), a specific PI3K blocker [51,52], or BAPTA (10 mM), a strong Ca^2+^ chelator, in the pipette solution. However, neither the blocker nor the chelator altered the paclitaxel-induced inhibitory effect (Figure 4). Deletion of PIP2 by phospholipase C (PLC) is a main KCNQ channel-modulating mechanism [53], and A-kinase anchoring protein (AKAP) is another important modulator of KCNQ channels [54]. However, application of neither the PLC inhibitor U73122 (1 μM) nor the AKAP disruptor st-Ht31 (50 μM) attenuated the inhibition induced by paclitaxel (Figure 4).

### 3.4. Inhibiting KCNQ Channels In Vivo Induces PIPN-like Pathological Alterations

**Figure 5:**
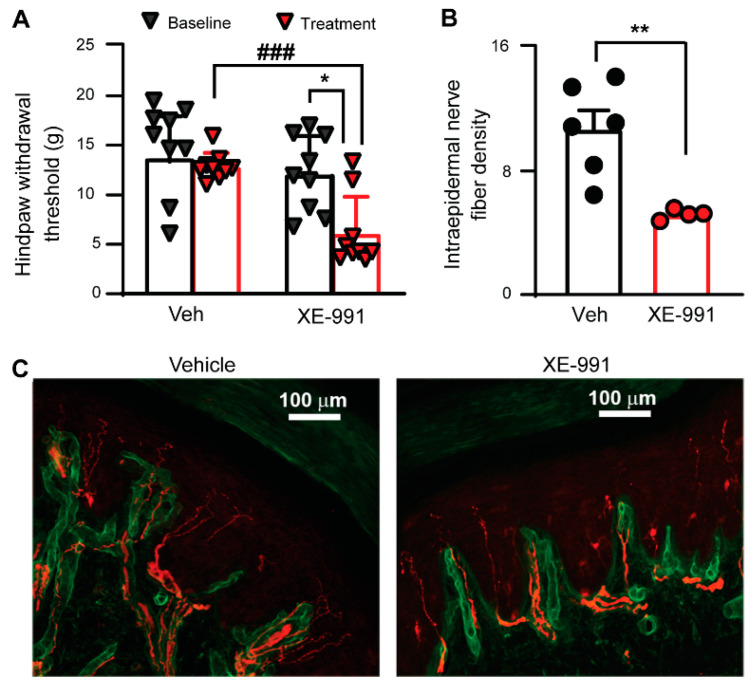
The effect of brief application of XE-991 (1 mg/kg, twice daily for 10 consecutive days) on the degeneration of IENFs and the development of chronic behavioral hypersensitivity. (**A**) Effects of XE-991 on the development of mechanical hypersensitivity in the hind paws (tested 3 weeks after the last XE-991 application). *p* = 0.5531, t(8) = 0.6191 for the vehicle group; *p* = 0.0107, t(8) = 3.309 for the XE-991 group, two-tailed paired Student’s *t* test. *p* < 0.0001, t(16) = 5.171 for postvehicle vs. postpaclitaxel, two-tailed unpaired Student’s *t* test. Colored symbols in columns represent one rat each. (**B**) The effect of XE-991 on skin IENF loss. Four to five sections from each rat were counted and averaged. Filled symbols in the bar graph represent individual rats. Unpaired *t* test (two-tailed). (**C**) Images showing rat skin IENFs (labeled with PGP9.5, red) that crossed the basal lamina (labeled with collagen IV, green) after XE-991 and vehicle treatment. Scale bars, 100 μm. Data are represented as mean ± SEM. *, *p* < 0.05; **, *p* < 0.01; ###, *p* < 0.001. Veh, vehicle.

To evaluate the role of KCNQ channels in the development of PIPN, we examined whether XE-991 could simulate paclitaxel-induced chronic alterations in naive SD rats. It has been reported that paclitaxel accumulation in DRGs remains detectable 10 days after the last injection [10]. We administered naive rats XE-991 (1 mg/kg, twice per day) or vehicle (saline, 1 mL, twice per day) for a prolonged period of ten consecutive days and tested for mechanical sensitivity by the *von Frey* test 3 weeks after the final injection. In vehicle-treated rats, the hind paw withdrawal threshold at 3 weeks after the last injection was not different from that obtained prior to the treatment; however, the hind paw withdrawal threshold of XE-991-treated rats was significantly decreased 3 weeks after treatment was ceased (Figure 5A). The thresholds for *von Frey* tests 3 weeks after final injection between the vehicle-treated and XE-99-treated groups were also significantly different with XE-991 group showing decreased thresholds (Figure 5A).

One of the hallmarks of PIPN is degeneration of peripheral nerves in the epidermal region [55]. We thus performed immunostaining on peripheral nerve fibers in glabrous skin from rats after behavioral tests. Specifically, we fluorescently labeled protein gene product 9.5 (PGP9.5, staining intraepidermal nerve fiber (IENF), red) and collagen IV (basal lamina, green) in glabrous skin sections and counted all PGP9.5-positive IENF fibers crossing the basal lamina. The IENF density in the XE-991-treated rats was significantly lower than that observed in the vehicle-treated group (Figure 5B–D). These data suggest that inhibiting KCNQ channel activity alone can induces PIPN-like peripheral neuropathy and mechanical hypersensitivity.

### 3.5. Paclitaxel Fails to Induce PIPN in KCNQ2 Conditional Knockout Mice

We next aimed to further assess the extent of involvement of peripheral sensory neuron KCNQ channels in the pathogenesis of PIPN. We hypothesized that deleting KCNQ2 from peripheral sensory neurons would eliminate most paclitaxel-induced pathological alterations.

**Figure 6:**
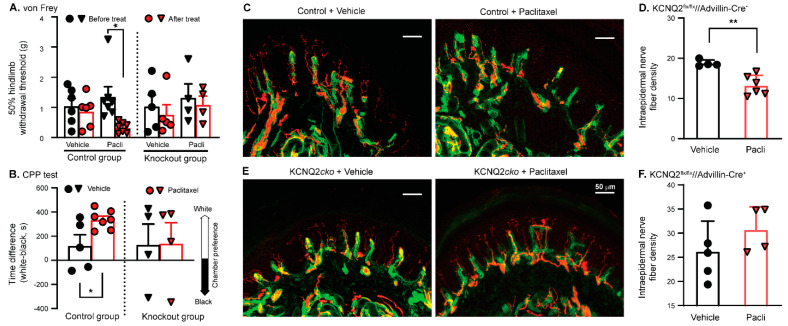
Effect of KCNQ2 deletion from primary sensory neurons on the development of PIPN. (**A**) Development of mechanical hypersensitivity in the hind paws (tested 3 weeks after final paclitaxel/vehicle application) of control and KCNQ2*cko* mice. Control group: *p* = 0.5814, t(5) = 0.5890 for vehicle; *p* = 0.0166, t(6) = 3.293 for paclitaxel, two-tailed paired Student’s *t* test. KCNQ2*cko* group: *p* = 0.2592, t(4) = 1.314 for vehicle; *p* = 0.3944, t(3) = 0.9918 for paclitaxel, two-tailed paired Student’s *t* test. Colored symbols in columns represent one mouse each. (**B**) Comparison of CPP responses between control and KCNQ2*cko* mice that received vehicle or paclitaxel injections. Animals were tested 4 weeks after the last injection. Symbols in columns represent individual mice (control and KCNQ2*cko*). *p* = 0.0252, t(10) = 2.629, unpaired *t* test (two-tailed) for control group; *p* = 0.9694, t(6) = 0.03997, unpaired *t* test (two-tailed) for KCNQ2*cko* group. *, *p* < 0.05; **, *p* < 0.01; Pacli, paclitaxel. (**C**) Images showing skin IENFs (stained with PGP9.5, red) that crossed the basal lamina (stained with collagen IV, green) in control mice receiving vehicle or paclitaxel injections. Scale bars, 50 μm. (**D**) Summary data showing the density of IENFs in the epidermis of control mice that did and did not receive paclitaxel injections. Filled symbols in the bar graph represent individual control mice. *p* = 0.0031, t(8) = 4.184, unpaired *t* test (two-tailed). (**E**) Images showing skin IENFs (red) that crossed the basal lamina (stained with collagen IV, green) in KCNQ2*cko* mice that underwent vehicle or paclitaxel injections. Scale bars, 50 μm. (**F**) Summary data showing the density of skin IENFs from KCNQ2*cko* mice that did and did not receive paclitaxel injections. Filled symbols in columns represent individual mutant mice. Data are represented as mean ± SEM. *p* = 0.2762, t(7) = 1.181, unpaired *t* test (two-tailed).

Both KCNQ2*cko* mice and their littermate controls received paclitaxel on days 1, 3, 5, and 7, for a total of four injections. Our data indicated that the paclitaxel-induced development of evoked pain tested by *von Frey* (Figure 6A) and spontaneous pain detected by CPP test (Figure 6B) was lower in Kcnq2 conditional knockout mice than in the littermate controls. While the difference in baseline (before paclitaxel treatment) mechanical behavioral sensitivity between the mutant and control groups was not significant, surprisingly, the baseline IENF density in KCNQ2*cko* mice was higher than that in the littermate controls. Furthermore, the IENF density in skin sections from the littermate control group was significantly impaired 4–5 weeks after treatment (Figure 6C,D), while that in sections obtained from paclitaxel-treated Kcnq2 conditional knockout mice was conserved (Figure 6E,F). Since selectively deleting KCNQ2 from primary sensory neurons greatly attenuates the PIPN pathological alterations, the data presented here suggest that KCNQ2 channels in primary sensory neurons play a critical role in initiating PIPN.

## 4. Discussion

Notably, patients who suffered acute pain during paclitaxel infusion usually had a high risk of developing PIPN [20,21], suggesting that paclitaxel-induced acute excitation of primary sensory neurons, especially nociceptors, underlies the pathogenesis of paclitaxel-induced peripheral neuropathy. Several studies have demonstrated that paclitaxel can induce an acute response in sensory neurons [23,56] and acute pain in rodents [57]. Our data indicated that the hyperexcitability of primary sensory neurons primarily resulted from paclitaxel-induced inhibition of KCNQ channels and that knocking out KCNQ2 from primary sensory neurons significantly attenuated the development of PIPN.

While we observed a correlation between the depolarization induced by paclitaxel and the KCNQ channel blocker XE-991, the latter has also been reported to block other channel types [58]. For example, 10 μM XE-991 has been demonstrated to inhibit Kv1.2 and Kv2.1 channel-mediated currents [58]. Both Kv1.2 and Kv2.1 are expressed in primary sensory neurons [59]; however, in our study, we noted that the specific Kv1 blocker α-DTX did not induce depolarization in some DRG neurons that responded to paclitaxel. Additionally, at the resting membrane potential of DRG neurons (−50 to −60 mV), Kv1.2 and Kv2.1 do not activate appreciably, as their activation threshold is approximately −45 to −40 mV [58]. We thus believe that the majority of the paclitaxel-induced depolarization of DRG neurons results from blockade of KCNQ channels.

We confirmed the above findings with experiments performed in DRG neurons dissociated from KCNQ2*cko* mice since paclitaxel failed to depolarize the majority of these cells. However, a few of these cells demonstrated XE-991-sensitive currents and paclitaxel-induced depolarization, which might have been due to inefficient expression of the Cre transgene in this subpopulation of cells and thus failure to delete KCNQ2 [60,61,62]. Alternatively, it is possible that other channels were involved in this residual response. It has been reported that paclitaxel inhibits Kv2.1 in H9c2 cells [63] and that Kv2.1 is expressed in DRG neurons [64,65]; however, as discussed above, the contribution from these ion channels should be minor, if at all present.

KCNQ channels are gated by PIP2 [45]. While our data indicated the involvement of PIP2 in the paclitaxel-induced inhibitory effect on KCNQ channels, the targeting of signaling pathways that potentially altered the local PIP2 levels—including the PI3K, PLC, NCS-1 signaling pathways—did not alter paclitaxel-induced current inhibition, despite reports indicating that some of these signaling pathways are modulated by paclitaxel [47,48]. Furthermore, decreasing PIP2 level only partially attenuates the paclitaxel-induced effect, suggesting that other mechanisms may be involved. We cannot exclude the possibility that paclitaxel directly binds to KCNQ channels to alter channel gating or channel interaction with/dependence on PIP2, as has been observed with zinc [66]. Further studies are warranted to determine this possibility.

While we did not observe detectable alterations in the mechanical sensitivity of Advillin-Cre-induced KCNQ2*cko* mice upon *von Frey* stimulation, the development of both mechanical allodynia and thermal hyperalgesia has been detected in Pax3-Cre-mediated KCNQ2*cko* mice [44]. In Pax3-Cre-mediated KCNQ2cko mice, however, no alteration in the resting membrane potential or firing threshold was observed in isolated DRG neurons, and the molecular components of myelinated fibers were well preserved [44]. Although DRG neurons from Pax3-Cre-mediated KCNQ2*cko* mice produced significantly more action potentials with less adaptation than did control neurons upon excitation, it is unknown to what extent such alteration in DRG neurons contributes to behavioral hypersensitivity. Unlike Advillin [67], Pax3 is indeed robustly expressed in brain tissue [44]. Thus, it is possible that deletion of KCNQ2 in the brain accounts for the hypersensitivity of both thermal and mechanical sensations observed in Pax3-Cre-mediated KCNQ2 mutant mice, based on the findings that (i) KCNQ channels are expressed in the spinal cord and brain [39,68] and (ii) intracerebroventricular injection of retigabine, a specific KCNQ channel activator [69], relieves inflammatory pain [70]. For the same reasons, KCNQ channels in the central nervous system could also at least partially contribute to repeated XE-991-induced chronic pain hypersensitivity. While mechanical sensation in Advillin-Cre-induced KCNQ2*cko* as assessed in behavioral tests did not change, an increased density of peripheral fibers was observed in this mouse line, which probably accounts, at least in part, for the compensatory alteration that was observed after KCNQ2 knockout. Such compensatory alterations usually cannot be induced by manipulating channels/proteins in the adult stage. Thus, inhibition of KCNQ channel activity alone in adult rats induced PIPN-like peripheral neuropathy, yet Advillin-Cre-induced KCNQ2*cko* displayed unaltered pain threshold.

Many molecules that are required for maintaining the function and structure of sensory fibers are generally considered to be synthesized in DRG neuronal cell bodies [71]. Sensory fibers that innervate hindlimb skin in humans are more than one meter in length, which is a long journey for transporting synthesized products from the somata to the periphery of DRG neurons [71]. Given that more than 90% of neuronal energy is used for maintaining membrane polarization during neuronal enhanced activity [72,73], the increased electrical activity of DRG neurons may result in rapid ATP depletion and peripheral fiber degeneration as proteins at the extremities fail to be replenished via energy-dependent transportation [74,75]. This effect could be especially obvious in unmyelinated nociceptors since unmyelinated axons consume approximately five-fold more metabolic energy than myelinated axons of the same volume [76]. In this regard, an acute loss of IENF was observed when capsaicin was applied to human skin [77], and prolonged systemic application of XE-991 induced PIPN-like alterations. In contrast, decreasing neuronal excitability by enhancing KCNQ channel activity during paclitaxel perfusion attenuated the development of PIPN [24]. There is high accumulation of paclitaxel in the DRGs but not in CNS tissue or ventral roots [10,78]. Accumulated paclitaxel thus inhibits KCNQ channels expressed in DRG neurons, resulting in consistent depolarization of these cells and the chronic alterations observed in PIPN. Thus, hyperexcitation of primary sensory neurons resulting from KCNQ channel inhibition after paclitaxel infusions, together with other proposed mechanisms [79], affects the longest sensory nerves to the extremities in a ‘stocking and glove’ distribution.

In this study, we used only male rats. While gender affects the gene regulation in primary sensory neurons after nerve injury [80], it has no obvious effect on the development of mechanical allodynia in rats following paclitaxel treatment [81]. The correlation between paclitaxel-induced acute pain and the risk of developing PIPN also occurs in female patients [21]. Such phenomena are consistent with what we observed in mouse in which no obvious behavioral and morphological discrepancies between sexes were detected (data not shown). Nevertheless, the molecular mechanisms we observed in male rats in this study will be further confirmed in female rats in future.

KCNQ channel activator, retigabine, significantly attenuates the development of PIPN [24]. However, the use of currently available KCNQ channel openers in the clinic is limited by their side effects. Flupirtine and retigabine show different side-effect profiles. While flupirtine causes liver injury, long-term application of retigabine is linked to reversible discoloration of the skin and eye [82]. The disparity in side-effect profiles suggests that it is the compounds themselves, but not KCNQ channels, that cause these side effects. Several ligands targeting KCNQ channels are under development and are offering promising features [83]. It is thus possible that more specific activators with fewer side effects can be available soon for PIPN treatment.

## Data Availability

The data that support the findings of this study are available from the leading corresponding author upon reasonable request.
